# To what extent are psychiatrists aware of the comorbid somatic illnesses of their patients with serious mental illnesses? – a cross-sectional secondary data analysis

**DOI:** 10.1186/s12913-017-2106-6

**Published:** 2017-02-23

**Authors:** Christina Dornquast, Juliane Tomzik, Thomas Reinhold, Matthias Walle, Norbert Mönter, Anne Berghöfer

**Affiliations:** 1 Institute for Social Medicine, Epidemiology and Health Economics, Charité – Universitätsmedizin Berlin, Luisenstrasse 57, 10117 Berlin, Germany; 2 IVPNetworks GmbH, Lübecker Str. 126, 22087 Hamburg, Germany; 3 PIBB GmbH & Co. KG – Psychiatrie Initiative Berlin Brandenburg, Tegeler Weg 4, 10589 Berlin, Germany

**Keywords:** Mental disorders, Somatic comorbidity, Secondary data analysis, Claims data

## Abstract

**Background:**

Somatic comorbidities are a serious problem in patients with severe mental illnesses. These comorbidities often remain undiagnosed for a long time. In Germany, physicians are not allowed to access patients’ health insurance data and do not have routine access to documentation from other providers of health care. Against this background, the objective of this article was to investigate psychiatrists’ knowledge of relevant somatic comorbidities in their patients with severe mental illnesses.

**Methods:**

Cross-sectional secondary data analysis was performed using primary data from a prospective study evaluating a model of integrated care of patients with serious mental illnesses. The primary data were linked with claims data from health insurers. Patients’ diagnoses were derived on the basis of the ICD-10 and the Anatomical Therapeutic Chemical (ATC) classification system. Diabetes, hypertension, coronary artery disease (CAD), hyperlipidaemia, glaucoma, osteoporosis, polyarthritis and chronic obstructive pulmonary disease (COPD) were selected for evaluation. We compared the number of diagnoses reported in the psychiatrists’ clinical report forms with those in the health insurance data.

**Results:**

The study evaluated records from 1,195 patients with severe mental illnesses. The frequency of documentation of hypertension ranged from 21% in claims data to 4% in psychiatrists’ documentation, for COPD from 12 to 0%, respectively, and for diabetes from 7 to 2%, respectively. The percentage of diagnoses deduced from claims data but not documented by psychiatrists ranged from 68% for diabetes and 83% for hypertension, to 90% for CAD to 98% for COPD.

**Conclusions:**

The majority of psychiatrists participating in the integrated care programme were insufficiently aware of the somatic comorbidities of their patients. We support allowing physicians to access patients’ entire medical records to increase their knowledge of patients’ medical histories and, consequently, to increase the safety and quality of care.

## Background

People with severe mental illnesses often have somatic comorbidities, meaning that one or more somatic diseases co-occur with the mental illness. In many cases, this phenomenon can be ascribed to a mutual dependence between the psychiatric illness and the somatic disease [1]. In fact, higher risks of getting a psychiatric disorder were shown for persons with somatic diseases [2]. On the other hand, also the risk of having a somatic disease is approximately twice as high for persons with severe mental illnesses than for people without mental disorders [3]. Thirty to fifty percent of patients experiencing psychiatric disorders have clinically relevant comorbid physical diseases [4], including cardiovascular disease, diabetes mellitus and respiratory and lung diseases [4–8]. In patients with severe mental illnesses, physical comorbidities often remain undiagnosed for a longer period of time than in those without severe mental illnesses [4, 9]. Many people with severe mental illnesses do not have or rarely consult a general practitioner and are confronted with various other barriers to receiving care [10–12].

Physicians working in the German health care system are not allowed to access patients’ health insurance data and do not have routine access to documentation from other providers of health care, in contrast to, e.g., UK and Denmark. To obtain information on comorbid diseases and other health care usage data, physicians must interview the patient or his/her relatives and actively order medical notes from colleagues in other specialties. Physicians need to rely on the completeness of the information given by the patient. This increases the risk that psychiatrists are not aware of comorbidities in patients with severe mental illnesses, which is problematic, e.g., when prescribed psychotropic drugs interact with drugs prescribed for somatic diseases by other physicians [13–16].

In this paper, we focus on psychiatrists’ awareness of their patients’ somatic comorbidities. We use data from a German pre-post study that evaluated the costs of a model of integrated health care for patients with serious mental illnesses [17]. The integrated health care programme comprises complex outpatient treatment provided by, e.g., psychiatrists and psychiatric nurses, general practitioners, clinicians and social workers. Inclusion in the programme substituted for an otherwise necessary hospitalisation. The study reported insufficient documentation of common somatic diseases by the treating psychiatrists. The purpose of this article was to compare the presence of selected somatic diseases in patients with severe mental illnesses with the frequency of documentation of these diseases by the treating psychiatrists. The selected somatic diseases were diabetes, hypertension, coronary artery disease (CAD), hyperlipidaemia, glaucoma, osteoporosis, polyarthritis and chronic obstructive pulmonary disease (COPD).

## Methods

### Study design

We performed a cross-sectional secondary data analysis based on a prospective, multi-centre, non-controlled observational study of an integrated care model in mental health [17]. The study was approved by the institutional ethics committee of the Charité–Universitätsmedizin Berlin.

### Study population and data sources

All patients who were insured by DAK-Gesundheit health insurance or a health insurance company belonging to the group BKK VAG Mid-Germany and who were newly included in the integrated health care programme between July 2007 and December 2009 in Berlin/Brandenburg and Lower Saxony were eligible to participate in this study. Detailed inclusion and exclusion criteria are reported elsewhere [17]. Briefly, patients were included in the integrated health care programme if they had an ICD-10 diagnosis within the range of F0.X to F8.X, showed seriously impaired social functioning as defined by a Global Assessment of Functioning (GAF) score of less than or equal to fifty, exhibited a severity score greater than or equal to five on the Clinical Global Impression (CGI) Scale and were at least 18 years old. Persons rated by the treating psychiatrist as having acute suicidal tendencies or a high likelihood of discontinuing treatment were excluded; the former were directly referred to inpatient treatment [17].

**Fig. 1:**
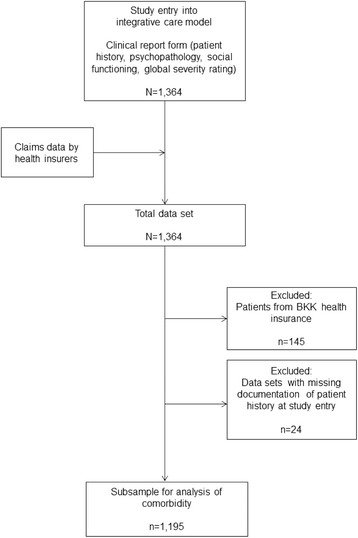
Study design and data management

From a sample of 1,364 patients in the integrated care model, we created a subsample for the present analysis by excluding patients with BKK statutory health insurance (*n* = 145) due to lack of permission to use their data as well as patients without existing clinical report forms from their treating psychiatrist and health insurance company data (*n* = 24) (see Fig. 1). The analysed subsample contained 1,195 participants. Psychiatrists’ clinical report forms were available from July 2007 to December 2009. In contrast, health insurance data were available from 31 July 2003 (first entry) to 31 December 2009 (last entry).

Psychiatrists documented relevant study information on medical history and the clinical status of their patients in clinical report forms containing socio-demographic data, medical history, ICD-10 codes for current psychological and somatic diagnoses, GAF scores, CGI scores and specific psychopathological scales. The GAF is a numeric scale that evaluates the psychological, social and occupational functioning of adults [18]. The CGI scale describes the severity of an illness [19]. The main psychiatric diagnosis, which was documented by the psychiatrists, was defined as being the one that resulted in the patient’s inclusion in the outpatient integrated care programme. Completeness of documentation was achieved as much as possible by a standardised query system used by the study personnel. If psychiatrists did not list any somatic comorbidities on a patient’s clinical report form, we assumed that no somatic comorbidities were known to them.

### Deriving ICD-diagnoses from ATC-Codes

The health insurance data contributed for each study participant comprised all Anatomical Therapeutic Chemical (ATC) classification codes for medications prescribed to the patient and ICD-10 codes if the patient was hospitalised, stayed at a rehabilitation centre or received an illness certificate. Somatic diagnoses were deduced either directly from the ICD-10 codes or indirectly from the medication regimen, as proposed by previous studies [20–24]. For our analysis, we selected highly prevalent somatic diseases with clinical relevance to patients with severe mental illnesses [1, 4, 25–27] or with an increased risk of severe interactions with common medications used for the treatment of severe mental illnesses [4, 13–16]. The selected somatic diseases were diabetes, hypertension, coronary artery disease (CAD), hyperlipidaemia, glaucoma, osteoporosis, polyarthritis and chronic obstructive pulmonary disease (COPD). Their respective ICD-10 codes and ATC codes for specifically prescribed drugs are listed in Table 1. Somatic diseases that could not unequivocally be deduced from the ATC codes of the prescriptions, such as stroke, epilepsy or Parkinson’s disease, were not considered, although they may be highly prevalent.

**Table 1:** Predefined criteria for selected somatic diseases using ICD-10 and ATC codes for prescribed medications^a^

Disease	ICD-10 code in health insurance data	ATC code prescription documented in health insurance data
Diabetes	E10-E14, G6320	A10 *(at least one time)*
Hypertension	I10-I15	C02 *(at least twice within 90 days)* OR at least two different ATC codes [C02, C03, C07, C08 or C09] *(within 90 days)*
CAD	I20-I25	*No distinct diagnosis by means of ATC codes*
Hyperlipidaemia	E780-E785	C10 *(at least one time)*
Glaucoma	H40, H42	S01E *(at least twice within 90 days)* AND manual review by a physician to identify patterns and decide individually
Osteoporosis	M80-M82	At least two ATC codes [G03XC01, H05, M05BA or M05BB] *(within 90 days)*
Polyarthritis	M05-M09	At least three ATC codes [M01B, M01CA, M01CB, M01CX, M02, L04AA13, L04AB01, L04AB04 or L04AX03] *(within 180 days)* AND manual review by a physician to identify patterns and decide individually
COPD	J44, J4200, J4390	At least three ATC codes [R03, R05] *(within 180 days)*

^a^proposed by previous studies [20–24]

### Assessment of psychiatrists’ knowledge

For each selected disease, we counted the number of patients for whom psychiatrists documented the respective diagnosis, as well as those for whom a diagnosis was derived on the basis of the health insurance data. Percentages were calculated by dividing the respective numbers by *N* = 1,195. To assess psychiatrists’ knowledge of their patients’ somatic diseases, the frequency of documentation of somatic diseases was descriptively compared using both psychiatrists’ clinical report forms and health insurance data for the following three steps of analysis:

First, we determined the number of somatic diagnoses documented by psychiatrists that were also derived from the health insurance data. Second, we determined the number of diagnoses that were documented by psychiatrists but not documented in the health insurance data. Third, we determined the number of diagnoses reported solely in the health insurance data.

### Sensitivity analysis

We performed a sensitivity analysis to verify the validity of our defined ATC code criteria for the detection of diseases. For each of the selected diseases, we investigated how many patients with an ICD-10 diagnosis were also identified by the defined ATC codes.

A descriptive statistical analysis was performed using R version 3.0.0 [28].

## Results

### Baseline characteristics

Table 2 shows the baseline characteristics of the study population. A total of 1,195 patients experiencing a psychiatric disorder, 69% of whom were females, were included in the study. The mean patient age at entry into the integrated care model was 48.0 ± 16.1 years. More than half of the patients had an affective disorder as their main psychiatric diagnosis. Schizophrenia, schizotypal and delusional disorders were documented as the main diagnoses in approximately one fifth of the patients.

**Table 2:** Baseline characteristics of the study population

*N* = 1,195	Number	Percent
Age (mean ± SD)	48.0 ± 16.1	
Sex		
Male	366	31%
Female	829	69%
Main psychiatric diagnosis^a^		
F0 Organic, including symptomatic, mental disorders	57	5%
F1 Mental and behavioural disorders due to psychoactive substance use	16	1%
F2 Schizophrenia, schizotypal and delusional disorders	234	20%
F3 Mood [affective] disorders	681	57%
F4 Neurotic, stress-related and somatoform disorders	137	12%
F6 Disorders of adult personality and behaviour	28	2%
F8 Disorders of psychological development	1	0%
Information missing	41	3%
Clinical Global Impression (CGI^b^) (mean ± SD)^a^	5.3 ± .7	
Global Assessment of Functioning (GAF^c^) (mean ± SD)^a^	36.7 ± 8.8	

^a^These numbers are from the first quarter of inclusion in the integrated health care programme

^b^Possible scores for CGI range from 1 to 7, with higher scores indicating more severe illness

^c^Possible scores for GAF range from 0 to 100, with higher scores indicating better psychological, social and occupational functioning

### Psychiatrists’ awareness of their patients’ comorbidities

The health insurance data contained 4,891 ICD-10 code entries and 96,951 ATC code entries for the 1,195 patients, of which 112 (2%) and 4,973 (5%), respectively, were missing, i.e., an insurance number and/or a date was listed without an ICD-10 or ATC code.

The number of patients for whom a diagnosis was documented by the psychiatrist and the number of patients who were diagnosed on the basis of the health insurance data differed widely (see Table 3). The frequency of documentation in the psychiatrists’ clinical report forms was very low, with hypertension (4%) and diabetes (2%) being the most commonly documented diseases. The frequency of documentation of the remaining diseases ranged from 0 to 1%. When combining data from health insurers and the psychiatrists’ clinical report forms, hypertension was the most frequently diagnosed disease (23%), followed by hyperlipidaemia (13%), COPD (12%) and diabetes (7%). Distinctly lower frequencies were observed for glaucoma (3%), CAD (3%), osteoporosis (2%), and polyarthritis (1%). Of all the patients in this study, 728 (61%) had no somatic comorbidities, and 272 (23%), 123 (10%) and 72 (6%) had one, two or three or more somatic comorbidities, respectively.

**Table 3:** Frequency of documentation and psychiatrists’ awareness of patients’ somatic illnesses

*N* = 1,195	Diagnosis documented by psychiatrist		Diagnosed in health insurance data^a^		Documented by psychiatrist and/or diagnosed in health insurance data^a^		Diagnosis documented by psychiatrist and documented in health insurance data^a^ (analysis step 1)		Diagnosis documented by psychiatrist and not documented in health insurance data^a^ (analysis step 2)		Diagnosis not documented by psychiatrist and documented in health insurance data^a^ (analysis step 3)	
	N	%	N	%	N	%	N	%	N	%	N	%
Diabetes	27	2%	82	7%	85	7%	24	28%	3	4%	58	68%
Hypertension	46	4%	254	21%	273	23%	27	10%	19	7%	227	83%
CAD	3	0%	30	3%	31	3%	2	7%	1	3%	28	90%
Hyperlipidaemia	5	0%	156	13%	157	13%	4	3%	1	1%	152	97%
Glaucoma	3	0%	38	3%	39	3%	2	5%	1	6%	36	92%
Osteoporosis	7	1%	24	2%	28	2%	3	11%	4	14%	21	75%
Polyarthritis	2	0%	8	1%	10	1%	0	0%	2	20%	8	80%
COPD	3	0%	138	12%	138	12%	3	2%	0	0%	135	98%

^a^According to Table 1

When comparing diagnoses documented by psychiatrists and diagnoses derived from health insurance data, fewer than 30% of diagnoses were reported in both data sources (see Table 3). This percentage was less than 10% for hypertension, CAD, hyperlipidaemia, glaucoma, polyarthritis and COPD. Patients for whom a diagnosis was documented by a psychiatrist but not documented in health insurance data were infrequent (less than 10% of the diagnosed patients). The majority of diagnoses (68 to 98%) were identified in the health insurance data but not documented by psychiatrists.

### Sensitivity analysis

We investigated how many patients with an ICD-10 code in their health insurance data were also diagnosed by ATC codes. For the majority of diseases, 52 to 75% were diagnosed by both codes (see Table 4). However, none of the four cases of polyarthritis diagnosed by ICD-10 code could be identified by the ATC criteria.

**Table 4:** Sensitivity analysis

*N* = 1,195	Diagnosed by ICD-10 code (in health insurance data)	Patients with somatic diagnoses derived from ATC criteria who also had documented ICD-10 codes in the health insurance data (in %)	
	N	N	%
Diabetes	12	8	67%
Hypertension	48	25	52%
CAD	0	n.a.^a^	
Hyperlipidaemia	0	0	0%
Glaucoma	5	3	60%
Osteoporosis	4	3	75%
Polyarthritis	4	0	0%
COPD	19	10	53%

^a^CAD patients were solely diagnosed by ICD-10 codes

## Discussion

Our analysis showed that psychiatrists’ documentation of somatic comorbidities in their patients was very low. The percentage of diagnoses that were documented in health insurance data but not by psychiatrists ranged from 68% for diabetes to 98% for COPD. This constitutes a problem, as many patients within our selected study population also suffer from somatic diseases. The number of patients with documentation of the selected somatic diseases ranged from 1% for polyarthritis to 23% for hypertension.

One of the main limitations of this study was the fact that relevant diagnoses could be missed in the health insurance data, as shown by our sensitivity analysis (see Table 4). However, we only had access to ICD-10 codes reported to a health insurer from hospitalisations, stays at rehabilitation centres and illness certificates. Data from the Association of Statutory Health Insurance - accredited Physicians (Kassenärztliche Vereinigung), which also contain ICD-10 codes originating from the outpatient sector, as well as data from general practitioners, were not available. Instead, we derived somatic diagnoses from the ATC codes of prescribed drugs. This method is more prone to omission and misclassification of disease. Various diseases are difficult or impossible to identify on the basis of ATC codes, e.g., CAD, heart failure and pain syndromes.

We may have underestimated the frequency of somatic diseases in this sample because a proportion of the patients studied may not have been treated for their somatic diseases and therefore would not have ATC codes in their health insurance data, and some patients were missing data from their health insurer. We expect that either technical problems or changes in health insurers are responsible for missing data, and hence we assume that selection bias is not a problem. Another limitation of our study is that, for certain patients, specific ATC codes were documented at the end of the period for which data were available. These patients were likely not assigned a diagnosis because our definition of diagnoses required several documentations of ATC codes within a certain time period. Additionally, the different time frames of the health insurance data and psychiatrists’ documentation obtained must be mentioned. However, these different time frames do not represent a serious constraint, as the selected somatic diseases are all chronic or lifelong diseases.

Our work is also limited by the fact that we could not discern why so many diagnoses were not documented by psychiatrists. It is possible that a psychiatrist was aware of a disease but did not document it in the clinical report form. However, this is unlikely due to the elicitation of this information in the context of a study, and a careful query process used to request non-completed answers.

Our data stem from a specialised treatment setting. However, the quality of documentation for the included patients is expected to be even better, due to the research setting in which it was gathered, than it would be in a typical treatment setting. The quality of the routine data from the health insurance company that were added to the primary data did not differ between patients inside or outside integrated care models, as the documented data are largely stipulated by the Germany Social Insurance Code.

In conclusion, we do not see this as a limitation to the validity of our results.

Previous studies have shown that many somatic diseases in patients with severe mental illnesses remain undetected or undiagnosed by their psychiatrists. A study conducted in 1989 reported that psychiatrists documented comorbidities in their patients in only 53% of cases [9]. In our analysis, diabetes was the only disease to be consistently documented by psychiatrists. This may be because it is particularly important for psychiatrists to know that their patients have been diagnosed with diabetes, as atypical antipsychotics have a diabetogenic effect. Additionally, patients usually have a diabetic ID card that can be shown to the psychiatrist. Such ID cards comprise amongst others information about the patients’ prescription schedule, dates of routine medicals checks and current blood levels of laboratory indicators. Furthermore, diabetes is a highly prevalent disease, and psychiatrists may be more aware of this disease and may ask about it more frequently.

Patients with severe mental illnesses are reported to be less likely to self-report their medical conditions to a physician. They are also less likely to report that their treating physician informed them about a co-occurring somatic disease [29].

Finally, the frequency of documented somatic diseases in our study population differed from those documented by other studies. The CATIE-study from 2005 reported a prevalence of hypertension of 27% for patients with schizophrenia [30], and hypertension was the most frequently diagnosed cardiovascular comorbidity in these patients. We found similar results in our sample with hypertension as most frequent documented disease (23%). Additionally, the authors of the CATIE-study reported a prevalence of diabetes of 13% in patients with schizophrenia, which is almost twice as high as the frequency of documentation we calculated [30]. The frequency of somatic diseases we calculated is expected to be lower because patients with other serious mental illnesses were included in addition to patients experiencing schizophrenia. Those patients are often treated with atypical antipsychotics, which are known to have diabetogenic effects. Other studies reported similar prevalences as the CATIE-study [25, 31]. A study from 2004 reported a prevalence of COPD of 23% in patients with severe mental illnesses [32]. This proportion is almost twice as high as the frequency we calculated in our sample (12%). This discrepancy is most likely explained by the fact that we derived diagnoses from the ATC codes of prescribed drugs. We were therefore more likely to miss relevant diagnoses.

## Conclusions

Many patients experiencing mental illnesses also have somatic diseases. These comorbidities pose challenges to the management of the mental, medical and personal health of a patient. Our data show that psychiatrists participating in the integrated care programme were insufficiently aware of the somatic comorbidities of their patients with psychiatric disorders. It can be assumed that outside of a research setting the level of awareness may be even lower.

Improved communication among physicians with distinct specialties is desirable to provide optimal therapy for a patient. However, the implementation of improved communication should not be the responsibility of the physician alone. Physicians have heavy workloads and may not be able to initiate an exchange of information with colleagues who are treating the same patient. Improved communication should be initiated and promoted by administrative institutions through the use of financial incentives or facilitated access to patients’ treatment data (e.g., through the use of an electronic health card). We support the idea of allowing physicians to access patients’ entire medical records to increase their knowledge of patients’ medical history and, consequently, to increase the safety and quality of care.

Psychiatrists should also be aware that the treatment of somatic diseases can affect psychiatric diseases and a patient’s response to psychiatric treatment. Hence, psychiatrists should obtain a thorough somatic medical history in their patient interviews. Because patients experiencing psychiatric disorders often have significant somatic comorbidities, and because of the associated health and economic consequences, additional studies, such as interview-based assessments of psychiatrists’ awareness of somatic comorbidities with the verification of true and false positives by independent assessors, are needed to investigate this issue.

## Abbreviations

ATC: Anatomical Therapeutic Chemical
CAD: Coronary artery disease
CGI: Clinical Global Impression
COPD: Chronic obstructive pulmonary disease
GAF: Global Assessment of Functioning
